# A modified Blumgart method using a homemade crochet needle facilitates pancreaticojejunostomy in laparoscopic pancreaticoduodenectomy: a retrospective cohort study

**DOI:** 10.1186/s12893-023-02308-9

**Published:** 2024-01-13

**Authors:** Bo Zhou, Zhenzhen Gao, Yang Tian, Sheng Yan

**Affiliations:** Department of Hepatobiliary and Pancreatic Surgery, The Second Affiliated Hospital, Zhejiang University School of Medicine, Hangzhou, 310003 China

**Keywords:** Laparoscopic pancreaticoduodenectomy, Blumgart method, Postoperative Complications, Pancreaticojejunostomy, Pancreatic fistula

## Abstract

**Background:**

Among the safest procedures for anastomosis in pancreaticoduodenectomy, Blumgart pancreaticojejunostomy is associated with low rates of postoperative pancreatic fistula (POPF) and postoperative complications. However, this technique is difficult to perform during laparoscopic pancreaticoduodenectomy (LPD). This study presents a modified Blumgart method using a homemade crochet needle to facilitate laparoscopic pancreaticojejunostomy and evaluates its safety and reliability.

**Methods:**

From February 2019 to October 2022, 96 LPD surgeries with the new technique were performed by the same surgeons in the Second Affiliated Hospital of Zhejiang University School of Medicine. The operative details (operative time, pancreaticojejunostomy time, POPF rate, postoperative complication rate, mortality rate) were analyzed along with clinical and pathological indicators (pancreatic duct diameter, pancreatic texture, and histopathological findings).

**Results:**

There were 54 men and 42 women with a mean age of 63.38 ± 10.41 years. The intraoperative bleeding volume, operative time and postoperative length of hospital stay were 198.43 ± 132.97 mL, 445.30 ± 87.05 min and 13.68 ± 4.02 days, respectively. The operation time of pancreaticojejunostomy was 66.28 ± 10.17 min. Clinically relevant POPFs (grades B and C) occurred in 14.6% of patients. Only one patient had postoperative abdominal hemorrhage and was cured after reoperation. There were no operative or in-hospital deaths. With our proposed modification, the pancreatic duct and jejunal orifice are aligned correctly during duct-to-mucosa (DTM) after the application of external traction through the homemade crochet needle. The space between the posterior wall of pancreatic remnant and jejunal loop can be exposed by adjusting the tension of the external threads, which can facilitate DTM.

**Conclusions:**

A modified Blumgart method using a homemade crochet needle could be technically feasible and safe during LPD. A randomized control trial is needed to confirm these findings.

## Background

Since laparoscopic pancreatoduodenectomy (LPD) was first reported in 1993 [1], LPD has been increasingly adopted worldwide for the treatment of benign and malignant tumors surrounding the duodenum, ampulla, lower common bile duct and head of the pancreas [2]. However, bleeding, abdominal infection, and even potentially life-threatening pancreatic fistula remain as existing challenges [3]. Several studies have demonstrated that the postoperative pancreatic fistula (POPF) rate remains high, with a related mortality rate of 3–8%; thus, POPF is known as the Achilles’ heel of the Whipple procedure [4–6]. A variety of approaches to reduce the incidence of POPF due to transanastomotic stenting, fibrin glue use, pancreaticogastrostomy and pancreaticojejunostomy (PJ) have been developed by pancreatic surgeons [7–9]. However, none of these approaches can completely avoid POPF.

Recently, it has been reported that Blumgart anastomosis, a well-accepted procedure among pancreatic surgeons, reduced the incidence of POPF by enhancing the adhesion between the pancreatic parenchyma and intestine [10, 11]. However, precise needle handling and prevention of suture tangling during LPD are still needed. We herein introduce our modified Blumgart approach to secure these structures with adjustable adhesions using a homemade crochet needle in LPD.

## Methods

### Study population

We performed the modified Blumgart PJ technique using a homemade crochet needle for LPD in February 2019 at the Department of Hepatobiliary and Pancreatic Surgery, The Second Affiliated Hospital, Zhejiang University School of Medicine. The inclusion criteria were resectable benign and malignant tumors surrounding the duodenum, ampulla, lower common bile duct and head of the pancreas. The exclusion criteria were malignant tumors with distant metastases and invading the superior mesenteric vessels on preoperative radiologic evaluation. We applied the enhanced recovery after surgery (ERAS) pathway in the LPD from December 2017, whereas the standard perioperative care protocol was used before. We retrospectively collected clinicopathologic variables (sex, age, body mass index (BMI), pancreatic texture, pancreatic duct diameter, and histopathological findings), operative details (total operating time, time needed for PJ, and intraoperative bleeding volume), and postoperative hospitalization data (amylase level on postoperative days 3 and 5, incidence of postoperative complications, incidence of clinically relevant postoperative pancreatic fistula (CR-POPF including grades B and C pancreatic fistula), hospitalization length, and 90-day mortality rate). All laparoscopic procedures were performed by the same surgeons following the same criteria and using the same anastomosis technique. This study was approved by the Ethics Review Committee of the Second Affiliated Hospital of Zhejiang University School of Medicine.

### Surgical procedures

#### Patient position and trocar distribution

**Fig. 1:**
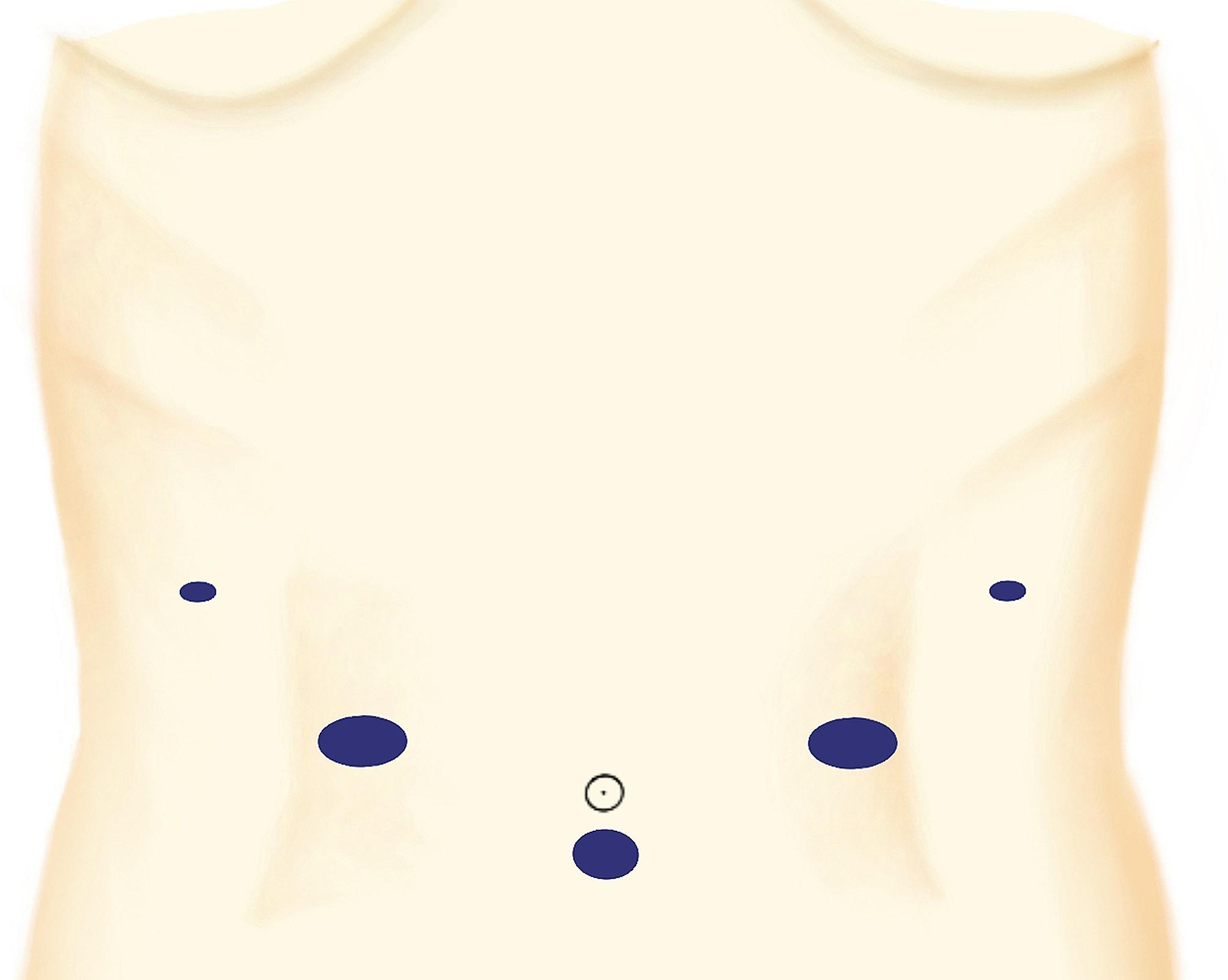
Placement of the trocars for LPD.

A supine position was adopted for the patient, with his or her legs spread and head elevated above the feet (at a 30-degree incline). The resection was performed with five trocars (Fig. 1): two 12-mm trocars (right and left upper quadrants), two 5-mm trocars (the right and left flank patterns) and one 10-mm trocar (umbilical).

#### Self-made crochet needle

**Fig. 2:**
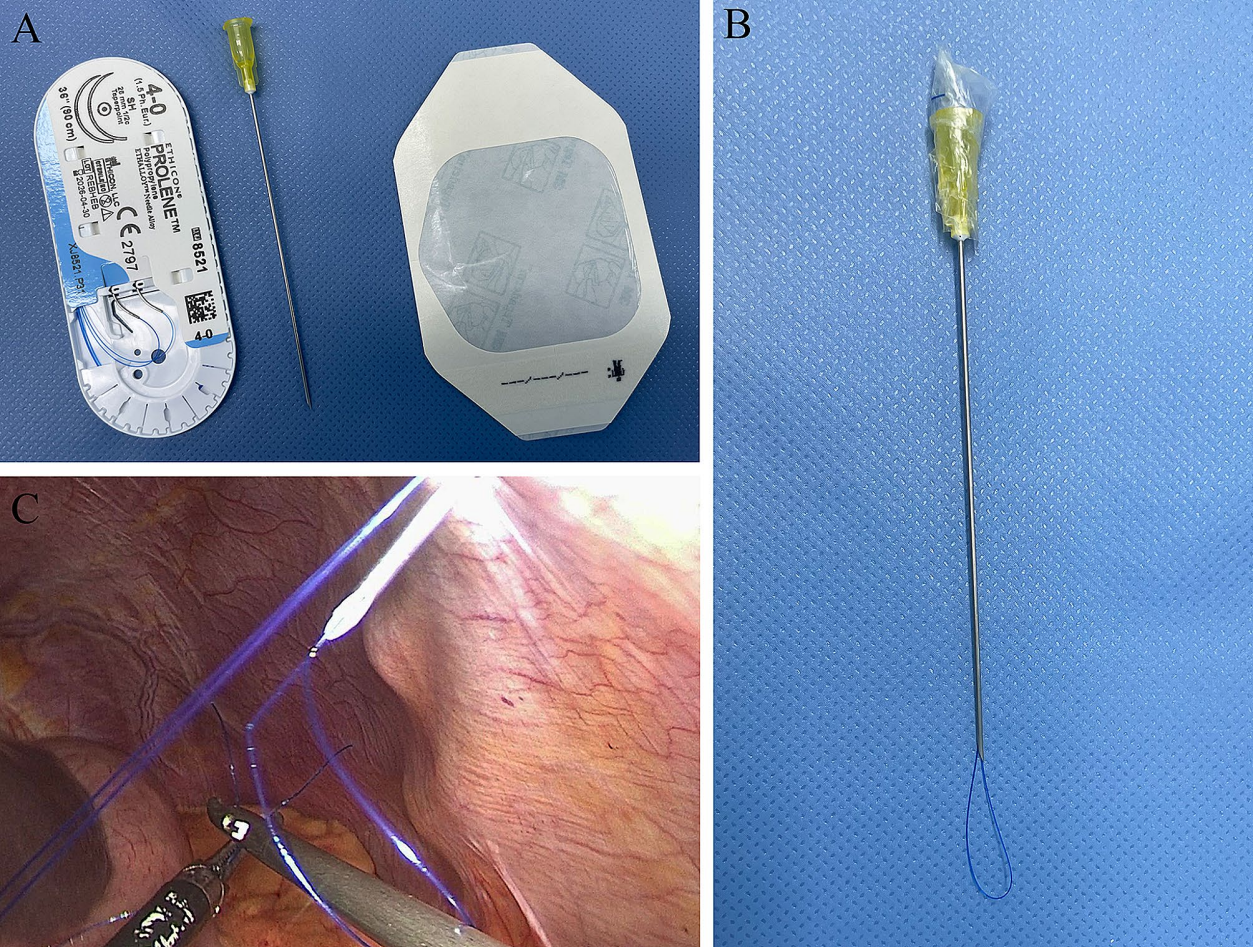
The details of the homemade crochet needle. The components of the homemade crochet needle (**A** and **B**). A U-shaped suture was created to encompass the posterior wall of the pancreatic parenchyma and the jejunal seromuscular layer and was fixed outside the body by a homemade crochet needle (**C**)

Our homemade crochet needle consists of 4 − 0 prolene (Ethicon Inc, Somerville, NJ, USA), an injection needle with a diameter of 0.9 mm and a length of 80 mm (Zhejiang Kindly Medical Equipment Co., Ltd., China) and a frame constructed with 3 M Tegaderm transparent film (3 M company, USA). First, the two ends of the prolene thread were passed through the injection needle and fixed at the tail of the injection needle using a 3 M Tegaderm transparent film dressing. Finally, a closed circle with a circumference of approximately 3 cm was formed on the tip of the injection needle (Fig. 2). During our operation, the posterior wall of the two pancreaticojejunostomy U-shapes was fixed outside the body using a homemade crochet needle to adjust the tension at any time and reduce interference from the threads under laparoscopy, especially in obese patients.

### PJ procedure

#### Preparation of the pancreatic stump and jejunal loop

**Fig. 3:**
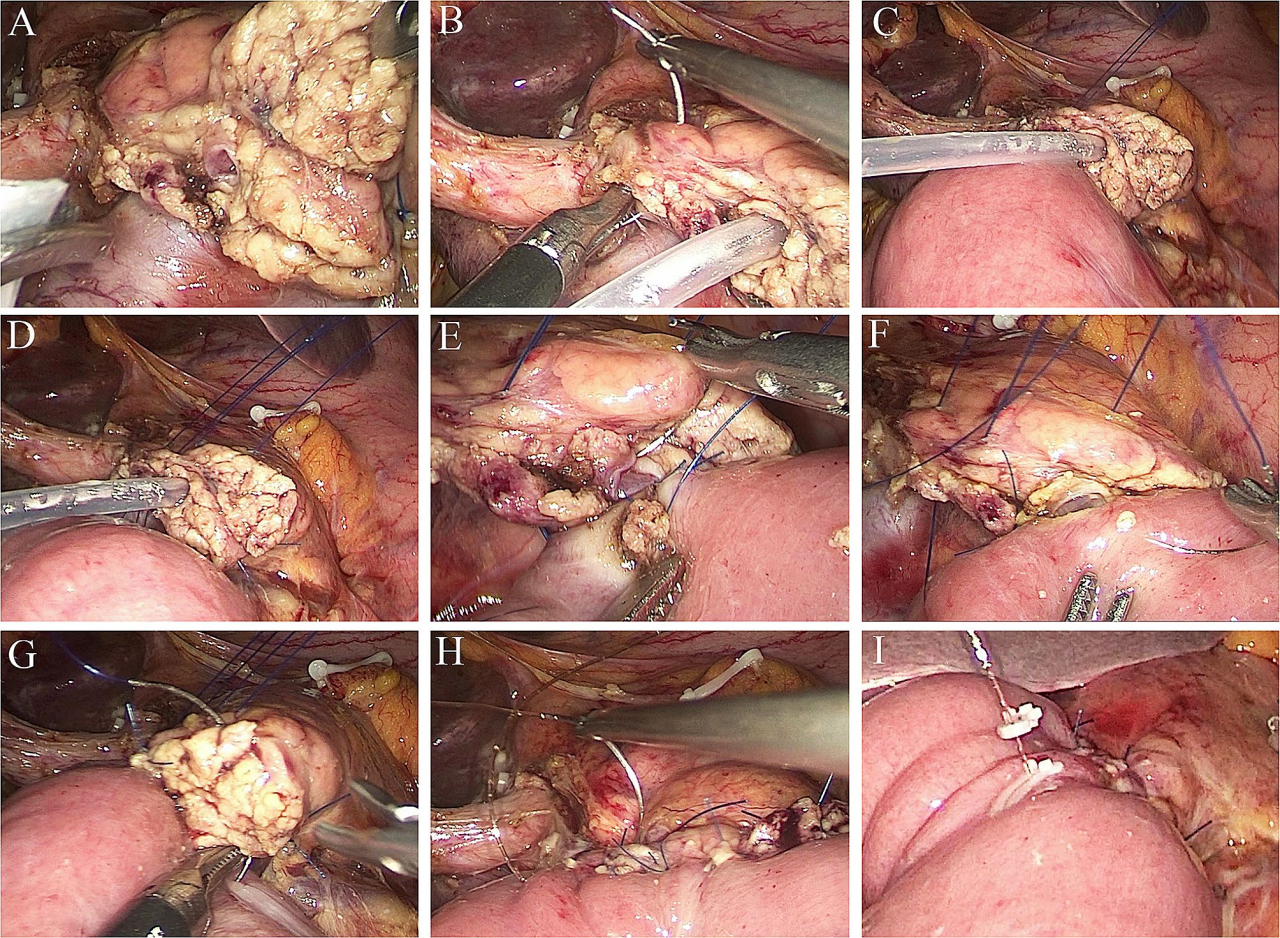
Intraoperative images of the modified Blumgart pancreaticojejunostomy procedure. (**A**) The specimen was removed first, and then pancreaticojejunostomy was performed. (**B**) A large 4 − 0 needle penetrated the pancreas 1 cm from the edge of the pancreatic stump. (**C** and **D**) A U-shaped suture was created to encompass the posterior wall of the pancreatic parenchyma and the jejunal seromuscular layer and was fixed outside the body by a homemade crochet needle. (**E** and **F**) The pancreatic duct and jejunal mucosa were sutured together with an internal pancreatic stent. (**G**) A third U-shape suture was placed between the pancreatic parenchyma and the jejunal seromuscular layer. (**H**) A single layer of continuous sutures was placed between the pancreatic stump and the anterior seromuscular layer of the jejunum using the 3/0 barbed suture Stratafix. (**I**) Final image after pancreaticojejunostomy

Typically, dissociation of 1–2 cm of the pancreatic stump borders was needed to perform PJ later (Fig. 3A). The main pancreatic duct was verified by either performing careful visual inspection for thick ducts or using a slender tube for narrow ducts. The closer end of the jejunum loop was closed. The jejunal limb was moved to the right of the middle colic vessels in a retrocolic fashion, while the blind end was placed close to the pancreatic remnant.

#### Pancreaticojejunal anastomosis

**Fig. 4:**
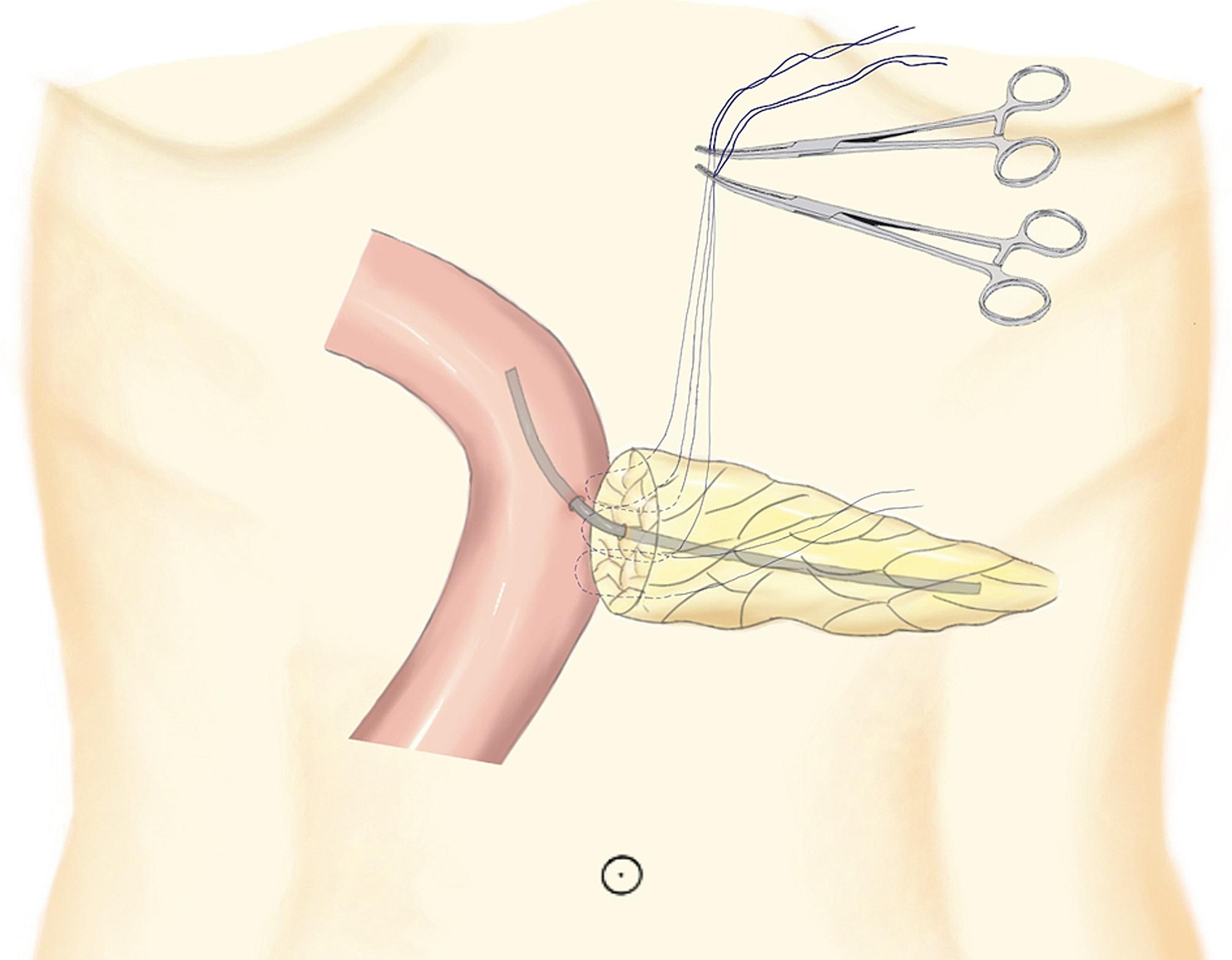
Two U-shaped transpancreatic stitches (one on side of the pancreatic duct and another encompassing the main pancreatic duct) were externalized with our homemade crochet needle, and the two threads were fixed with a vascular clamp to facilitate adjusting the tension between the pancreas and jejunum

A large 4 − 0 prolene suturing needle was used to vertically enter the pancreas stump 1 cm from the ventral side, extending out from the dorsal side of the edge of the pancreas (Fig. 3B). After the needle has been inserted horizontally along the long axis of the jejunum, it is advanced 1 cm within the seromuscular layers of the jejunum. Next, the needle protrudes 1 cm from the pancreatic dorsal side to the ventral side. A U-shaped suture was created, and the needle was cut. Then, both ends of the thread were lifted out of the body with our homemade crochet needle, and the two threads were fixed with a vascular clamp to facilitate adjusting the tension between the pancreas and jejunum (Fig. 3C). Furthermore, a similar second U-shaped suture encompassing the main pancreatic duct that extended between the pancreatic parenchyma and the jejunal seromuscular layer was created (Fig. 3D), and both ends of the thread were also lifted out of the body with our homemade crochet needle (Fig. 4). Finally, a third U-shaped suture was created with both ends of the thread and fixed with a hemolock (Fig. 3G). This maneuver has an advantage over the classical technique because at this point, the posterior faces of both the jejunum and pancreas are not yet sutured so the duct-to-mucosa (DTM) anastomosis can be made.

The posterior semicircle sutures of the DTM were placed at the 4, 6, and 8 o’clock positions using 5 − 0 PDS II (Ethicon Inc, Somerville, NJ, USA) (Fig. 3E). With traction on the externalized transpancreatic stitches, the jejunal loop can be reinserted into the pancreatic posterior face, while the stitches on the posterior face of the TMD served as anchors for the loop. It is usually necessary to place a stent into the pancreatic duct. Later, stitches were placed on the anterior face at the 10, 12, and 2 o’clock positions of the DTM in the same manner (Fig. 3F). Once the DTM anastomosis was completed, the three U-shaped sutures were knotted sequentially. Then, a single layer of continuous sutures was made between the pancreatic stump and the anterior seromuscular layer of the jejunum using the 3/0 barbed suture Stratafix (Ethicon Inc, Somerville, NJ, USA) (Fig. 3H and I).

After PJ was completed, biliary and gastric reconstructions were sequentially performed. Two external drainage tubes were routinely placed around the hepaticojejunostomy and PJ.

#### Postoperative management

The drain output was recorded each day after the operation. The amylase level in the drainage fluid was measured on postoperative days 3 and 5 and at any time when POPF was suspected. One abdominal CT scan was routinely conducted on postoperative day 5. To prevent infection after surgery, broad-spectrum antibiotics and anti-anaerobic drugs were used for 72 h postoperatively. All patients received octreotide after the operation to decrease the volume of pancreatic external secretion. POPF was diagnosed and graded according to the definition from the International Study Group on Pancreatic Fistula (ISGPF) (2016 version). If the amylase content of any measurable drainage on or after postoperative day 3 was greater than 3 times the upper normal serum value or the drains were either left in place for > 3 weeks or repositioned endoscopically or percutaneously, a grade B POPF was considered. On the other hand, grade C POPFs were those that needed reoperation or led to single/multiple organ failure and/or mortality. The postoperative complications included delayed gastric emptying, abdominal hemorrhage, gastrointestinal anastomotic hemorrhage, bile leakage, infection, chylous fistula and mortality. On postoperative days 5, if the amylase content of any measurable drainage is lower than 3 times the upper limit of normal serum amylase and follow-up abdominal CT shows no sign of abdominal fluid in the operative region, the drainage tube can be removed.

### Statistical analysis

Student’s t test was performed to compare continuous variables represented as mean ± standard deviation (SD). Categorical data are described as numbers (percentages) and were compared using the chi-square test or Fisher’s exact test. Furthermore, the differences between non-POPF group and POPF group were evaluated by t tests in the case of normally distributed variables or by chi-square test in the case of categorical data. Statistical significance was determined by a P value of 0.05. All analyses were conducted with SPSS 22.0 software (SPSS, Inc., Chicago, IL, USA).

## Results

### Demographic and clinicopathological features of patients

There were 96 patients who underwent LPD with the new technique, including 54 men and 42 women with a mean age of 63.38 ± 10.41 years (Table 1). The average BMI of the patients was 22.52 kg/m^2^. The leading indication for LPD was pancreatic ductal adenocarcinoma (n = 48, 50%), followed by duodenal papillary carcinoma (n = 21, 21.9%), distal cholangiocarcinoma (n = 12, 12.5%), ampullary carcinoma (n = 7, 7.3%), intraductal papillary mucinous neoplasm (n = 4, 4.2%), solid pseudopapillary tumor (n = 2, 2.1%), pancreatic neuroendocrine tumor (n = 1, 1.0%), and duodenal adenoma (n = 1, 1.0%). The average total operative time, intraoperative blood loss and postoperative hospital stay were 445.30 min, 198.43 mL and 13.68 days, respectively. The mean operation time for PJ was 66.28 min. Grade B POPF occurred in 13 patients (13.5%), while 1 grade C POPF was observed. One patient with postoperative abdominal hemorrhage was cured after reoperation to achieve homeostasis. Six patients (6.3%) suffered from chylous fistula, 4 patients (4.2%) suffered from delayed gastric emptying, 3 patients (3.1%) suffered from pneumonia, 2 patients (2.1%) suffered from bile leakage, 2 patients (2.1%) suffered from abdominal infection, and 1 patient (1.0%) suffered from gastrointestinal anastomotic hemorrhage, which were all treated with conservative therapy. There were no operative or in-hospital deaths.

**Table 1:** Baseline characteristics and results of patients who underwent LPD

	LPD (n = 96)
Age (years)	63.38 ± 10.41
Sex (M/F)	54/42
BMI (kg/m^2^)	22.52 ± 2.87
Pathology	
Ampullary carcinoma	7 (7.3%)
Distal cholangiocarcinoma	12 (12.5%)
Pancreatic ductal adenocarcinoma	48 (50%)
Duodenal papillary carcinoma	21 (21.9%)
Duodenal adenoma	1 (1.0%)
Neuroendocrine tumor	1 (1.0%)
Pancreatic duct stones or pancreatitis	0
Intraductal papillary mucinous neoplasms	4 (4.2%)
Solid pseudopapillary tumor	2 (2.1%)
Operative time (min)	445.30 ± 87.05
Pancreaticojejunostomy time (min)	66.28 ± 10.17
Vascular resection (portal vein reconstruction)	1 (1.0%)
Blood loss (mL)	198.43 ± 132.97
Pancreatic parenchymal texture	
Soft	51
Hard	45
Pancreatic duct diameter	
> 3 mm	35
≤ 3 mm	61
Grade B POPF	13 (13.5%)
Grade C POPF	1 (1.0%)
Reoperation	1 (1.0%)
Delayed gastric emptying	4 (4.2%)
Bile leakage	2 (2.1%)
Abdominal hemorrhage	1 (1.0%)
Gastrointestinal anastomotic hemorrhage	1 (1.0%)
Abdominal infection	2 (2.1%)
Pneumonia	3 (3.1%)
Chylous fistula	6 (6.3%)
Postoperative hospital stay (day)	13.68 ± 4.02
Mortality < 90 days	0

LPD, laparoscopic pancreaticoduodenectomy; BMI, body mass index; POPF, postoperative pancreatic fistula

### Comparisons between the non-POPF and POPF subtypes

When the patients in these subgroups were compared (Table 2), the incidence of soft pancreas was higher in the POPF group than in the non-POPF group (P = 0.016). Furthermore, the incidence of pancreatic ductal adenocarcinoma or pancreatitis was lower in the POPF group than in the non-POPF group (P = 0.013). However, no significant differences were observed in age (P = 0.094), BMI (P = 0.575), operative time (P = 0.419), intraoperative bleeding (P = 0.610) or pancreatic duct diameter (P = 0.270). The fistula risk score of non-POPF group was significant lower than that of POPF group (3.18 ± 1.69 vs. 5.50 ± 1.34, P < 0.001).

**Table 2:** Comparisons between the non-POPF and POPF subtypes following LPD

	POPF (n = 14)	None POPF (n = 82)	P value
Age (years)	68.60 ± 7.34	62.78 ± 10.57	0.094
BMI (kg/m^2^)	22.04 ± 2.68	22.58 ± 2.90	0.575
Operative time (min)	466.50 ± 71.53	442.84 ± 88.70	0.419
Blood loss (mL)	225.00 ± 211.15	195.35 ± 169.19	0.610
Pancreatic parenchymal texture			**0.016**
Soft	12	42	
Hard	2	40	
Pancreatic duct diameter			0.270
> 3 mm	3	30	
≤ 3 mm	11	52	
Pathology			**0.013**
Pancreatic ductal adenocarcinoma or pancreatitis	3	47	
Others	11	35	

LPD, laparoscopic pancreaticoduodenectomy; BMI, body mass index; POPF, postoperative pancreatic fistula. The bold values in the table denote P values less than 0.05 (indicating a significant difference)

## Discussion

In recent decades, LPD has been adopted in many medical centers for the radical treatment of both benign and malignant pancreatic and periampullary disease. To date, the mortality and morbidity of LPD have significantly declined, and the data show that patients who underwent LPD in high-volume centers achieved a better prognosis than those treated in low-volume centers [12–15]. However, the overall postoperative morbidity rate remains high, and the most fatal complication is POPF. The POPF rate in recent literature ranges from 10 to 29% [16, 17], and this complication can also prolong patient hospitalization, increase mortality and increase costs. Some studies have revealed various risk factors for POPF, such as the PJ technique, pancreatic texture, and duct diameter. Moreover, many efforts have been made to decrease POPF incidence. In fact, many improvements to the PJ technique have been developed to minimize the rate of POPF.

End-to-end and end-to-side approaches are the main approaches to PJ after pancreatoduodenectomy (PD) in most medical centers. Peng et al. reported a study that consisted of 150 patients who underwent PJ, with a 0% rate of POPF [18]. Although this method achieved the lowest POPF rate, it has not been repeated in subsequent foreign studies. Maggiori et al. reported a POPF rate of 36% using Peng’s technique [19]. Furthermore, duct-to-mucosa anastomosis has been improved in various ways. Karavias et al. reported a POPF rate of 7.9% after using their method called true duct-to-mucosa anastomosis. Although it was not mentioned in the report, the PJ time seemed prolonged because mucosal eversion was performed [20]. Interestingly, triple-layer duct-to-mucosa PJ was introduced by Su et al. with a POPF rate of 4%. The three layers included the pancreatic duct to jejunal mucosa, the pancreatic capsular parenchyma to the jejunal seromuscular and the pancreatic capsular parenchyma to the jejunal serosa [21]. However, despite the numerous PJ techniques, no prospective randomized controlled trials (RCTs) have been carried out to determine the best approach.

Recently, Blumgart anastomosis, a new DTM anastomosis procedure well accepted among pancreatic surgeons, was reported to reduce the incidence rate of POPF [10, 11]. However, this pancreatic anastomosis procedure has disadvantages that mainly limit the extent of LPD. First, multiple small sutures must be left untied, causing confusion in the field of vision. Second, it is difficult to create the posterior face of the DTM anastomosis when the posterior face’s capsular stitches have been previously tied [22–24]. Therefore, we applied a modified Blumgart method using a homemade crochet needle to facilitate PJ in LPD. With our proposed modification, the pancreatic duct and jejunal orifice are aligned correctly during DTM after the application of external traction through the homemade crochet needle. The space between the posterior wall of pancreatic remnant and jejunal loop can be exposed by adjusting the tension of the external threads, which can facilitate DTM. Moreover, there are few small sutures left untied, which makes the surgical field clearer. Finally, the homemade crochet needle does not leave scars after puncture and can be used for puncture at many places on the abdominal wall. In the present study, the CR-POPF rate (grade B-C) was only 14.6%, and 1 grade C POPF was observed using our PJ procedure; these rates are lower than those in most reported studies. Only one patient with postoperative abdominal hemorrhage was cured after reoperation, thereby achieving homeostasis. There were no operative or in-hospital deaths. Thus, this new PJ procedure is feasible for achieving a safe LPD.

This study has several potential limitations due to its retrospective design. It is necessary to perform a prospective, randomized study that includes more patients and centers in order to validate the rate of pancreatic fistula following this type of anastomosis.

## Conclusions

We demonstrate the feasibility and safety of a modified Blumgart method using a homemade crochet needle to facilitate PJ in LPD. However, randomized controlled trials are needed to further verify the feasibility of the present PJ technique in LPD.

## Data Availability

The datasets analyzed during the current study are available from the corresponding author on reasonable request.

## Abbreviations

LPD: Laparoscopic pancreaticoduodenectomy
POPF: Postoperative pancreatic fistula
PJ: Pancreaticojejunostomy
BMI: Body mass index
CR-POPF: Clinically relevant postoperative pancreatic fistula
DTM: Duct-to-mucosa
SD: Standard deviation
